# Finite Element Analysis of Cervical Spine Kinematic Response during Ejection Utilising a Hill-Type Dynamic Muscle Model

**DOI:** 10.3390/bioengineering11070655

**Published:** 2024-06-27

**Authors:** Yikang Gong, Zhenghan Cheng, Ee-Chon Teo, Yaodong Gu

**Affiliations:** 1 Faculty of Sports Science, Ningbo University, Ningbo 315211, China; 2311040043@nbu.edu.cn (Z.C.); guyaodong@nbu.edu.cn (Y.G.); 2 Research Academy of Grand Health, Ningbo University, Ningbo 315211, China

**Keywords:** finite element, emergency ejection, neck motion, active muscle, Hill-type model

## Abstract

To determine the impact of active muscle on the dynamic response of a pilot’s neck during simulated emergency ejection, a detailed three-dimensional (3D) cervical spine (C0–T1) finite element (FE) model integrated with active muscles was constructed. Based on the Hill-type model characterising the muscle force activation mechanics, 13 major neck muscles were modelled. The active force generated by each muscle was simulated as functions of (i) active state ($N_{a}$), (ii) velocity ($F_{v}$(v)), and (iii) length ($F_{L}$(L)). An acceleration-time profile with an initial acceleration rate of 125 G·s^−1^ in the 0–80 ms period, reaching peak acceleration of 10 G, then kept constant for a further 70 ms, was applied. The rotational angles of each cervical segment under these ejection conditions were compared with those without muscles and with passive muscles derived from the previous study. Similar trends of segmental rotation were observed with S- and C-curvature of the cervical spine in the 150 ms span analysed. With active muscles, the flexion motion of the C0–C2 segments exhibited higher magnitudes of rotation compared to those without muscle and passive muscle models. The flexion motion increased rapidly and peaked at about 95–105 ms, then decreased rapidly to a lower magnitude. Lower C2–T1 segments exhibited less variation in flexion and extension motions. Overall, during emergency ejections, active muscle activities effectively reduce the variability in rotational angles across cervical segments, except C0–C2 segments in the 60–120 ms period. The role of the active state dynamics of the muscles was crucial to the magnitude of the muscle forces demonstrated. This indicates that it is crucial for pilots to consciously contract their muscles before ejection to prevent cervical spine injuries.

## 1. Introduction

In humans, the head and neck are the most flexible, exposed, and unprotected segments of the human body. It is the most vulnerable and frequent region of injury in any traumatic or unexpected situation. The head-neck system is a complex biomedical linkage with multiple degrees of freedom of movement [1,2,3] whereby various neck muscles are irritated and contracted (shortened or lengthened) coordinately in concert, either bilaterally or unilaterally, to control the head-neck movement in fulfilling various daily human activities and protecting the head-neck from injury. 

In high-speed activities such as punching in kickboxing [4], pilot emergency ejection [5,6], and passenger vehicular accidents [7], large magnitudes and different directions of impact acting on the head and neck commonly occur. Exceeding either the force or motion tolerance of the neck could cause a high risk of injury, leading to soft tissue injuries of the spine, cervical disc degeneration, ligament tears, muscle strains, and cervical bony fractures.

Specifically, with the advancements in high-performance aircraft technology, the airspeed of aircraft has increased tremendously, even beyond the speed of sound. In a total aircraft failure, the emergency exit ejection from the cockpit often subjects the pilot’s torso to high acceleration [6]. In this emergency, the exposed, unconstrained, and mobile head and neck complex would either flex or extend and compress; the severity of these motions often exposes the neck to a frequent site of injury in the spinal column, causing spinal cord injuries. This makes the safe ejection of crew members from disabled aircraft a crucial concern [6,8].

It is widely believed that muscles play a vital role in resisting such impact forces and providing support for the head. Teo et al. [9] developed a sophisticated C0–C7 cervical spine 3D FE model, validated using data from vertex drop tests, and investigated the effects of passive muscles in the response of the head-neck complex under simulated ejection scenarios. The findings revealed that passive neck muscles effectively reduced cervical segmental rotation and alleviated the maximum stress experienced by the intervertebral disc nucleus and annulus. However, the study did not deeply explore the role of active muscle activation. Another FE study by Li et al. [5] demonstrated that muscle pre-activation protects the neck during emergency ejections, while sustained muscle activity increases both the axial load and rotation angle on the neck, suggesting that the mechanisms and effects of active neck muscle contractions require further research.

Using FE models to simulate and understand the response of the head-neck structure under extreme dynamic conditions offers an innovative method. Compared to physical in vivo and in vitro experiments, this approach allows researchers to study biomechanical responses under various conditions without exposing subjects to risk [10,11]. 

Accordingly, in this study, a previously validated C0–C7 FE model data were modified to incorporate the active muscles’ biomechanical response characteristics and properties with the C7–T1 articulated segment, was developed and exercised to study the kinematic response of the head and neck in ejection. The computed cervical segmental rotation compared against those without muscles and with passive muscles was discussed. This study should provide new insights into neck muscle activity affecting the response of the cervical spine during ejection.

## 2. Materials and Methods

### 2.1. Modelling

In this study, an anatomically geometrically accurate 3D FE model that extensively includes the skull (C0) through to the first thoracic vertebra (T1) along with all its attached soft tissues was constructed. A brief, comprehensive explanation of the development process of the model [12] is provided. The model was built based on the 3D geometric data of a 68-year-old male cadaver. The coordinates of the bony surface profile of the skull (C0) and C1–T1 vertebrae were captured continuously in an orderly manner using a flexible digitizer (Faro Arm, Bronze Series, Faro Technologies, Inc., Lake Mary, FL, USA) and post-processed using ANSYS R16 (ANSYS, Inc., Canonsburg, PA, USA) to generate the FE meshed model. The associated soft tissues (ligaments and intervertebral discs (IVDs) and 13 muscles) were constructed based on the average geometric dimensions with their attachment points from published literature [13,14,15,16,17,18,19]. The final FE model consists of key structural components of each motion segment of the neck: cortical bone, cancellous bone, posterior structures, annulus fibrosus of the intervertebral disc, nucleus pulposus, endplates, major ligaments, and muscle groups crucial to the movement of the neck. The C7–T1 segment was added to allow for the attachment of the lower neck muscles in the ejection simulation analysis using ANSYS R16 with the LS-Dyna solver option.

**Figure 1:**
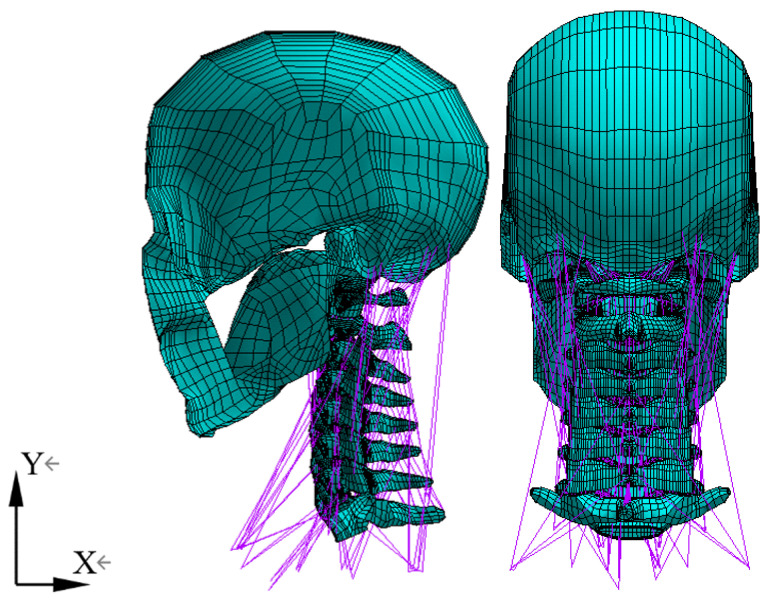
C0–T1 FE model with muscles.

Figure 1 shows the C0–T1 FE model, which consists of 27,712 elements and 31,749 nodes. In the model, different 2D and 3D element types with different mechanical and structural properties were assigned to the bony vertebrae, associated ligaments, and muscles according to data derived from literature [15,20,21,22,23,24] in the simulation analysis. 

#### Mathematical Modelling of Skeletal Muscles

**Figure 2:**
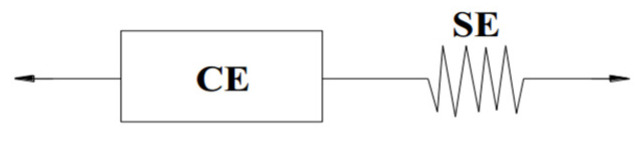
The Hill-type muscle model.

In this study, the first detailed model characterising the muscle activation mechanics introduced by Hill [25] in 1938 was adopted. This seminal Hill-type model, as shown in Figure 2, consists of two primary components: a contractile element (CE) responsible for generating active muscle force and a series elastic element (SE), often akin to tendon structures. 

The functional characteristics of the CE are described through three main functions: the force-length relationship ($F_{L}$), the force-velocity relationship ($F_{v}$), and the active muscle state ($N_{a}$); the activated muscle force is as shown in Equation (1) [26].

*F_CE_(t) = Na(t) F_L_(L) F_v_(V)* (1)

Based on Hill’s model, researchers have developed their own numerical models to predict muscle forces when simulating human body movements in clinical, sport, and occupational biomechanics studies [27,28,29,30,31]. The applications of this kind of muscle model in FE studies for the head-neck complex under simulated ejection impact are not common. In this study, specific variables based on the required format to define each active muscle as spring-damper elements were input. There are 10 variables, as shown in Table 1, defining the simulation of active muscles in the dynamic analysis.

According to Wittek’s study [32], for all cervical muscle groups, the values of *S_V_*, *F_pe_*, *L_max_* and *K_sh_* were set to 1.0, 0.0, 0.8 and 2.0, respectively (as shown in Table 1); other variables *L_o_*, *V_max_*, *F_max_*, and *L_max_* of the muscles groups used in this investigation are indicated in Table 2.

**Figure 3:**
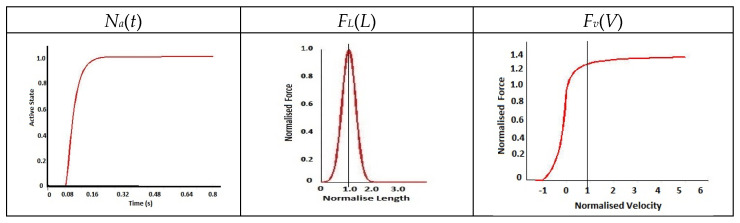
Graphs of functions *Na*(*t*), *F_L_*(*L*) and *Fv*(*V*).

Figure 3 shows the computed graphical plots of the three functions described by different mathematical equations with various constants derived from literature [26,32,33,34,35]. With the variables from Table 1 and Table 2, the active forces, [{*F_CE_*(*t*)}*n*, as defined in Equation (1)], of all muscles (*n*) with a reflex time of 80 ms are generated and simulated. 

### 2.2. Boundary and Loading Conditions

**Figure 4:**
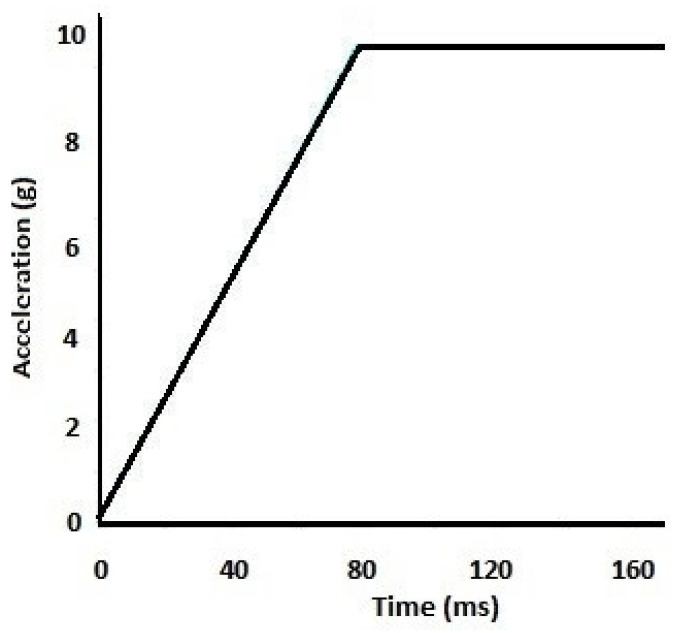
Input acceleration on T1 for simulated ejection.

In this study, the loading and boundary conditions were applied the same as in the previous study [9]. Acceleration-time curve, as shown in Figure 4, started from zero and accelerated at a rate of 125 G·s^−1^ for 80 ms, then kept constant at 10 G for a further duration of 70 ms and was applied to the inferior surface of T1 in Y-direction. The entire FE model was subjected to an ejection acceleration-time profile, restricted to move only in the Y-axis direction, with unconstrained C1–C7 vertebrae and C0. 

The computed various cervical levels’ segmental rotations in the sagittal plane were plotted and superimposed with previous results without muscles and passive muscles investigated under the same acceleration-time profile simulation [9] to explore the varying outcomes under ejection scenarios. 

## 3. Results

Based on the symmetrical modelling of the muscles about the sagittal plane, coupled with the defined loading and boundary conditions, the whole FE model was ejected vertically. Different magnitudes of segmental rotation in the sagittal plane at different times were captured; the secondary motions in the other two anatomical planes were not investigated.

**Figure 5:**
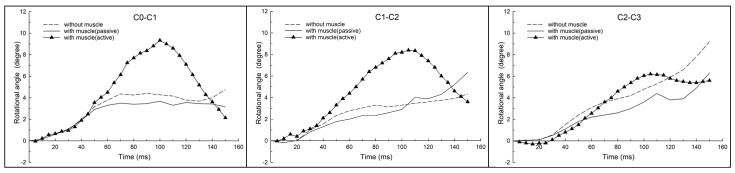

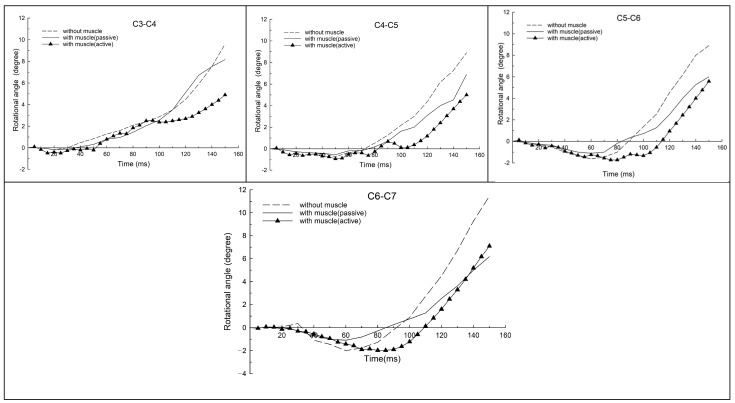
Cervical segmental rotational angles over time in ejection under different scenarios.

Figure 5 shows the various segmental rotational angles over time graphs of the cervical spine (simulated without muscles, with passive muscles and active muscles) in the ejection condition. The graphs primarily reflected flexion (+^o^) and extension (−^o^) motions, as applied in the previous study [9].

Under the active muscle scenario, within the range of 0–110 ms, the upper segments (C0–C2) experienced flexion rotation of increasing magnitude with time, and the lower segments (C2 to C7) experienced extension-flexion rotation of varying magnitudes with time. A longer duration of extension motion of the C5–C6 and C6–C7 segments was observed. During the 0–110 ms period, the entire C0–C7 structure formed an inverted S-shaped curvature, with upper segments flexed and lower segments extended. This inverted S-shaped curvature resulted in a forward C0 anterior translation phenomenon, where C0 translated anteriorly relative to C7 with limited extension rotation. After 110 ms, all cervical segments were in a state of flexion with an increasing magnitude of flexion angle of C0 relative to C7. Subsequently, the entire cervical spine was in a mirrored C-shaped curve.

For the C0–C7 model simulated without muscles and with passive muscles under ejection simulation analysis, the segmental rotations were plotted based on the validated C0–C7 FE model investigated [9]. Compared to the results simulated with active muscle conditions, for the time between 0 and 110 ms, similar trends of the flexion-extension-flexion motions of the same order of rotation magnitude, except in C0–C2 segments of lower magnitudes were observed, and the cervical spine exhibited an inverted S-curvature. After 110 ms, the whole cervical flexed, exhibiting a mirrored C-curvature.

For the C0–C2 segments, the flexion rotational angle increased continuously over time and peaked at 150 ms for conditions without muscles and with passive muscles. However, simulated with active muscles, flexion rotations of C0–C2 increased and peaked at about 100–105 ms, then decreased with time, exhibiting an inverted U-shaped trend.

**Figure 6:**
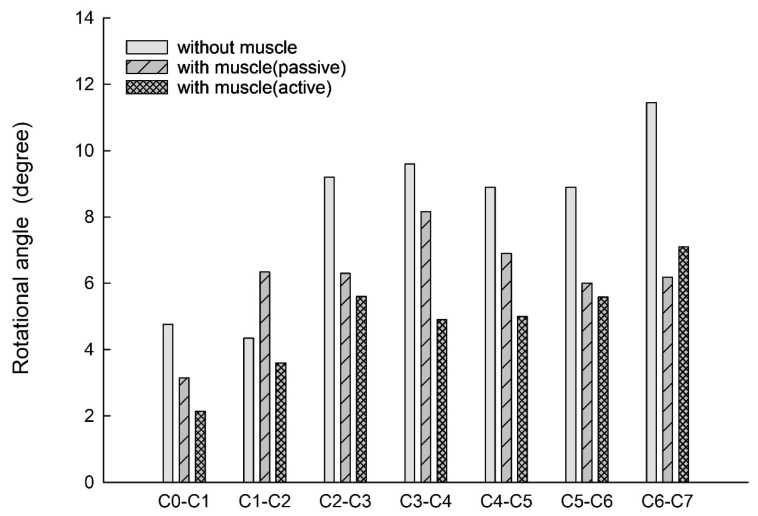
Segmental flexion rotation at 150 ms.

Figure 6 shows the bar chart plot of the peak flexion rotation at 150 ms for without, with passive and active muscles simulated. Compared to the condition with no muscles, the effect of passive and active muscles was demonstrated, and almost all segments showed a reduction in flexion rotation. When muscles are actively engaged, the greatest reduction of about 43.2% in the rotational angle at C1–C2, and the smallest reduction of about 7% at C5–C6 was observed, compared to those results with passive muscles. 

**Figure 7:**
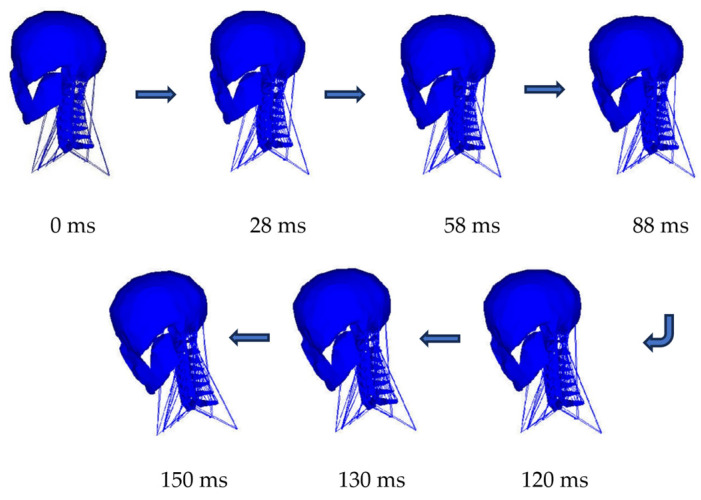
Vertical motion trajectory of C0–T1 FE model at different times.

Figure 7 shows the deformed plots of the head-neck under the action of active muscles in ejection simulation. In the solution run, the simulation of the ejection process for a period of 150 ms was set, and different deformed plots of the whole C0–T1 at different times were plotted.

## 4. Discussion

This study aims to explore the effect of active muscle engagement on the dynamic response of the neck during emergency ejection scenarios, utilising a comprehensive head-neck FE model. Incorporating a mathematical simulation of active muscle activities into an existing validated model [9]. Under drop impact simulation without muscles, the cervical spine exhibited either S-shaped or C-shaped curvatures, which correlated well with experimental data [36]. This validated model was exercised under ejection simulation without muscles and with passive muscles, which also exhibited both types of curvature. In the current study, a detailed anatomically accurate C7–T1 segment was meshed into this validated model [9], and Hill-type muscles describe the 13 active major neck muscles incorporated in the C0–T1 FE model.

With ejection simulation of 10G acceleration applied at the inferior surface of T1 in the Y-direction, post-processing of the results plotted graphically demonstrated inverted S-shaped and mirrored C-shaped curvatures of the cervical spine in the 150 ms time span. This revealed the reliability and validity of the C0–T1 FE model utilised in the current study.

At 150 ms, both active and passive muscle engagements significantly reduced the rotation angle compared to the no-muscle condition, consistent with previous research findings [9]. In previous studies [9], with passive muscles simulated compared to without muscle condition, the largest reduction of 46.8% was observed at the C6–C7 segment and the smallest reduction of 14.7% at the C3–C4 segment. However, with active muscle activation, the greatest reduction of 55% and the least reduction of 17% were observed at C0–C1 and C1–C2, respectively, compared to those without muscle condition. In the C0–C1 segment, the longus colli muscle significantly influences the anterior tilting angle of the spine. Its primary attachment points span extensively, from the base of the skull to the lower cervical spine, playing a crucial role in stabilising the head-neck region and facilitating forward head tilting. It is speculated that muscle activation tightens the longus colli, further effectively controlling the rotational angle of this segment. Multiple muscles, including the longus colli, anterior vertebral muscle, latissimus dorsi head, and semispinalis capitis head, are involved in the C1–C2 segment. The anterior vertebral muscle, a large deep cervical muscle that extends from the skull base to the third thoracic vertebra, primarily functions to stabilise and tilt the cervical spine forward. Despite covering multiple cervical segments, its impact on the C1–C2 segment remains significant. The slight variation in rotation angle might be due to the antagonistic action between the anteriorly located longus colli and anterior vertebral muscle, and the posteriorly positioned latissimus dorsi head and semispinalis capitis head. Further research is needed to elucidate the specific mechanisms of individual and collective muscle actions on the cervical spine.

Different segments experienced varying peak extension or flexion angular movements at different times after the ejection. In this study, during the time from acceleration onset to a period after acceleration stabilised (0–110 ms), mechanically, we stipulated that the inferior vertebral body of C7 was subjected to greater axial forces, compressing the neck. Due to the substantial axial forces, the lower cervical spine segments (particularly C3–C7) were compressed into extension movements, while the upper cervical spine segments were in a state of flexion due to freed articulated head flexion rotation. Thus, the entire C0–C7 construct formed an inverted S-shaped curvature with upper segments in flexion and lower segments in extension. After 110 ms, the lower cervical segments translated into flexion motion, and the entire cervical spine formed a mirrored C-shaped curvature. Indeed, such S-shaped and C-shaped curvatures of the cervical spine are also observed under other conditions [9,12,37].

**Figure 8:**
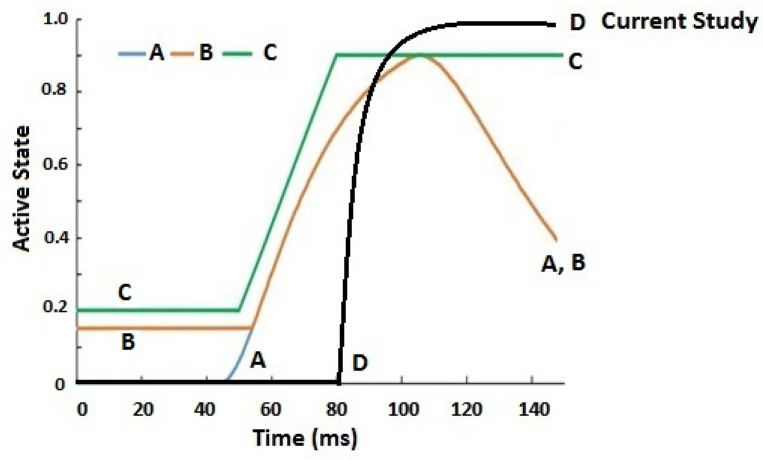
Graphs of active state applied in the current study and Li et al. [5].

In a study by Li et al. [5], ejection simulation of the C0–T1 FE model with three different muscle activation strategies (Na(t)) was investigated. Their results showed cervical segments (C1 to C6) were in a stage of flexion motion of increasing magnitudes with time; the lower segment (C6–C7) showed increased flexion rotation that peaked at about 80 ms, then decreased, exhibiting a flattened inverted U-shaped pattern. Overall, the whole cervical spine was flexed, exhibiting mirrored C-curvature. Compared to the current study, with a reflex time of 80 ms, the whole cervical spine exhibited flexed and extended rotations at different segment levels with time, exhibiting S- and C-curvatures. Specifically, the rotation angle of the C0–C2 segments showed an inverted U-shaped pattern, peaking around 100 ms. Figure 8 showed graphs of Na(t) from both studies, while Table 3 showed C1–C2 and C6–C7 segments’ rotation based on Li et al. [5] and the current study, plotted in bar chart form at 40, 80, 120, and 140 ms, suggesting the role of the active state dynamic of the muscles may be crucial to the magnitude of muscle forces.

Several other factors could account for these differences: First, the model parameters used varied, suggesting the need for future research to establish a more refined and representative head-neck FE model to minimise these discrepancies. Secondly, differences in the reflex time, mechanical and structural properties of all spinal components, and mathematical formulae with parameters in defining the active muscle force-time profiles (variations in the active force under the Hill muscle model) could have led to the differences shown in Table 3. Thirdly, all the materials defined in this analysis were idealised to be homogeneous, isotropic, and linearly elastics; however, studies by Karimi et al. [38,39] showed that as soft tissues are generally anisotropic and viscoelastic, their mechanical responses depend on the load rate, suggesting exploration in this direction. Future investigations were suggested to find the relationship between muscle activation reflex time and the deactivation of individual neck major muscles in the biomechanical response of the whole cervical spine under different dynamic impacts. Also from the study by Moldovan et al. [40], a new 3D technology together with the application of FE analysis in the field of traumatology could provide future direction to further improve the FE model and conduct comparative analysis.

## 5. Conclusions

This study established a head-neck FE model that includes the action of active muscles to analyse the impact of muscle activation on the dynamic response of the neck. By analysing the rotational angles of various spinal segments, it was concluded that during emergency ejection simulated with active muscles, similar trends of segmental motions of flexion and extension of different magnitudes compared to without muscle and with passive muscle conditions. contraction of neck muscles enhanced the stability of the pilot’s neck movements, reducing the risk of neck injury during emergency ejection. Inverted S- and mirrored C-curvatures of the cervical spine were exhibited under ejection simulation without passive and active muscle conditions. The rate of flexion rotation accelerated between 0 and 95 ms then decelerated at almost the same rate for the C0–C2 segments showing an inverted U-shaped pattern, peaking around 100 ms with a flexion angle of between 8 and 9 degrees, these sudden changes in the rotation might cause spinal injuries. Further study is suggested so that training related to spinal muscle control to limit such sudden changes in the rotation of the upper cervical spine.

## Figures and Tables

**Table 1:** Variables for describing active spring damper muscles.

S/N	Variable	Value	Descriptions
1	*L_o_*	-	initial muscle length
2	*V_max_*	-	the maximum contractile element shortening velocity
3	*S_V_*	1.0	scale factor for *V_max_* against active state
4	*A*	-	activation level-time function (*N_a_*(*t*))
5	*F_max_*	-	peak isometric force
6	*TL*	-	active tension-length function (*F_L_*(*L*))
7	*TV*	-	active tension-velocity function (*F_v_*(*V*))
8	*F_pe_*	0.0	force-length function for the parallel elastic element
9	*L_max_*	0.8	relative length when passive force *F_pe_* reaches *F_max_*
10	*K_sh_*	2.0	constant which governs the exponential rise of *F_pe_*

**Table 2:** Variables to model active muscles.

S/N	Muscle Groups	*L_o_* _(mm)_	*V_max_* _(mm/s)_	*F_max_* _(kg*mm/s_^2^_)_	A _(mm_^2^_)_
1	Sternocleidomastoid	180	1980	44,825	358.6
2	Longus colli	60	650	25,000	200
		15	165	25,000	200
		40	440	25,000	200
3	Longus capitis	85	935	50,000	200
		55	605	50,000	200
4	Scalenus anterior	110	1210	41,400	165.6
		80	880	41,400	165.6
5	Scalenus medius	65	715	10,900	43.5
6	Scalenus posterior	60	660	34,000	136
7	Trapezius	160	1760	87,500	350
8	Semispinalis capitis	120	1320	37,500	150
		90	990	37,500	150
9	Semispinalis cervicis	80	880	17,950	71.8
10	Longissimus capitis	115	1265	20,000	80
		75	825	20,000	80
11	Longissimus cervicis	75	825	20,000	80
12	Splenius capitis	175	1925	18,700	224.4
13	Splenius cervicis	110	1210	18,700	84.7

**Table 3:** C1–C2 and C6–C7 segmental rotation at different times.

	Segment	C1–C2	C6–C7
Times			
40 ms		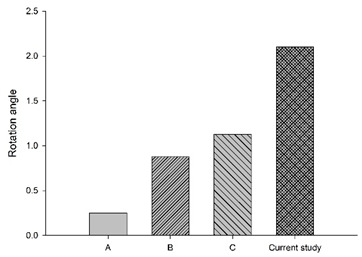	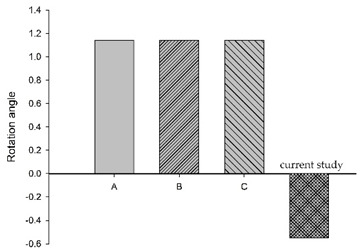
80 ms		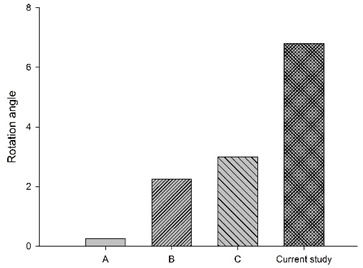	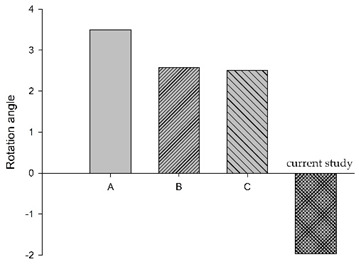
120 ms		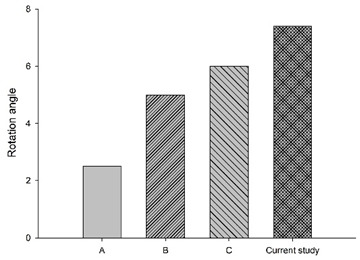	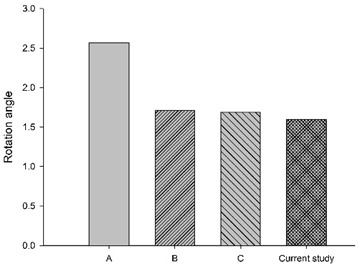
150 ms		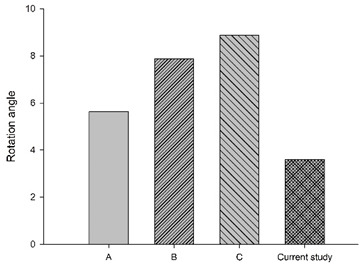	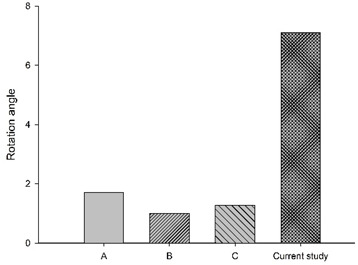

## Data Availability

The original contributions presented in this study are included in the article. Further inquiries can be directed to the corresponding authors.
